# Glucagon‐like peptide agonists: A prospective review

**DOI:** 10.1002/edm2.462

**Published:** 2023-12-14

**Authors:** Zamara Mariam, Sarfaraz K. Niazi

**Affiliations:** ^1^ Pharmaceutical Scientist, Inc. Chicago Illinois USA; ^2^ College of Pharmacy University of Illinois Chicago Illinois USA

**Keywords:** cancer, diabetes, GLP‐1, GLP‐2, glucagon, hormones, neurological disorders

## Abstract

**Background:**

Glucagon‐like peptide‐1 receptor agonists (GLP‐1RAs) have emerged as promising therapeutic options for addressing Type‐2 diabetes, obesity, and related conditions. Among these, semaglutide, tirzepatide, liraglutide etc., all notable GLP‐1RA, have gained attention owing to their favourable pharmacological properties and clinical efficacy.

**Aims:**

This comprehensive review aims to provide a detailed analysis of both the currently available GLP‐1RAs in the market and those undergoing clinical trials. The focus is on examining their mechanism of action, pharmacokinetics, efficacy in glycemic control and weight management, safety profile, and potential applications.

**Materials & Methods:**

The review employs a systematic approach to gather information on GLP‐1RAs. Relevant literature from the currently literature and ongoing clinical trials is thoroughly examined. Detailed scrutiny is applied to understand the mechanism of action, pharmacokinetic properties, and clinical outcomes of these agents.

**Results:**

The review presents a comprehensive overview of the GLP‐1RAs, highlighting their distinct mechanisms of action, pharmacokinetic profiles, and clinical effectiveness in glycemic control and weight management. Safety profiles are also discussed, providing a holistic understanding of these therapeutic agents.

**Discussion:**

The findings are discussed in the context of advancements in the field of GLP‐1RAs. Potential applications beyond diabetes and obesity are explored, shedding light on the broader implications of these agents in managing related conditions.

**Conclusion:**

In conclusion, this review underscores the significance of GLP‐1RAs, with a specific focus on semaglutide, in the management of type 2 diabetes, obesity, and beyond. By synthesizing information on their mechanisms, pharmacokinetics, efficacy, and safety, this review provides valuable insights into the potential benefits these agents offer, contributing to the ongoing discourse in the field.

## Graphical abstract

A picture showing how the AI‐driven translation is more relevant.

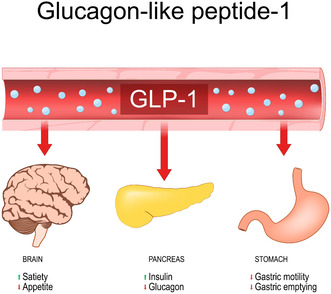

## 1 INTRODUCTION

### 1.1 Background

Glucagon is a hormone that works with other hormones and bodily functions to control glucose levels in the blood. It is made in the alpha cells of the pancreas, scattered in small clusters throughout the organ, known as the Islets of Langerhans.^1^ It is a single‐chain polypeptide composed of 29 amino acids (H‐His‐Ser‐Gln‐Gly‐Thr‐Phe‐Thr‐Ser‐Asp‐Tyr‐Ser‐Lys‐Tyr‐Leu‐Asp‐Ser‐Arg‐Arg‐Ala‐Gln‐Asp‐Phe‐Val‐Gln‐Trp‐Leu‐Met‐Asn‐Thr‐OH) and plays a crucial role in regulating blood glucose levels.^2^ The primary role of glucagon is to prevent blood glucose levels from dropping too low by promoting the conversion of stored glycogen (from liver cells) into glucose, which is then released into the bloodstream through glycogenolysis.^3^ When necessary, glucagon also promotes the synthesis of glucose from amino acids through gluconeogenesis. Thus, glucagon is an emergency product in diabetes to prevent hypoglycemia.^4^ In addition, glucagon has a role in signalling the body to break down stored fat (triglycerides) into fatty acids for use as energy in cells. This occurs when the body's glucose level is too low to meet its energy needs.^5^

Glucagon exerts its effects by binding to its specific receptor, the glucagon receptor (GCGR), primarily expressed in the liver and kidneys. A specific receptor for glucagon was inferred from physiological studies carried out in the mid‐20th century. Still, the gene encoding the glucagon receptor was not identified until modern molecular biology techniques were invented. The gene for the glucagon receptor (GCGR) was cloned in the 1990s, which allowed for a better understanding of its structure and function.^6^ This receptor belongs to a more prominent family of proteins known as G‐protein‐coupled receptors (GPCRs)^7^ that activate an intracellular protein called a G protein. This G protein, in its activated form, triggers the production of a second messenger molecule called cyclic AMP (cAMP) from ATP^8^ that activates a type of enzyme called Protein Kinase A (PKA). The PKA phosphorylates various target proteins, leading to several downstream effects. For instance, in the liver, PKA phosphorylates and activates an enzyme known as glycogen phosphorylase, which breaks down glycogen into glucose. PKA also inhibits an enzyme called glycogen synthase, preventing further synthesis of glycogen; both actions contribute to the release of glucose into the bloodstream.^9^, ^10^, ^11^

**FIGURE 1:**
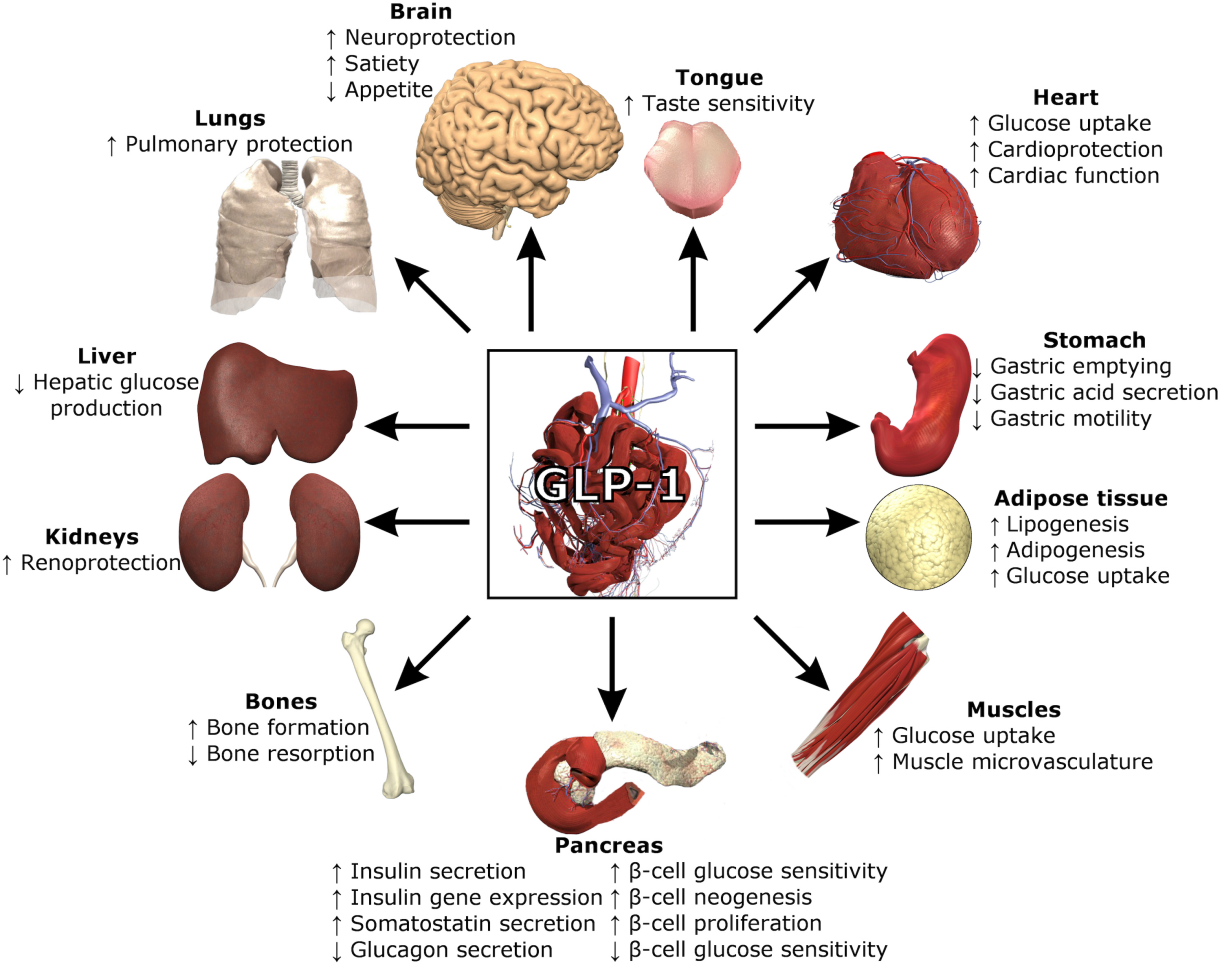
Functions of GLP‐1 [By Lthoms11—Own work, CC BY‐SA 4.0, https://en.wikipedia.org/w/index.php?curid=55236027].

The multiple functions of GLP peptides are shown in Figure 1.

### 1.2 Glucagon‐like peptides

Glucagon‐like peptide‐1 (GLP‐1) and glucagon‐like peptide‐2 (GLP‐2) are derived from the same proglucagon gene. The names reflect their genetic origin and similarity to glucagon and each other. The proglucagon gene is expressed in the alpha cells of the pancreas, L‐cells in the intestine, and certain neurons in the brain. In the pancreas, the proglucagon gene is processed to produce glucagon. However, the same proglucagon gene is processed differently in the intestinal L‐cells and neurons, producing GLP‐1, GLP‐2, and another peptide called glicentin.^12^

GLP‐1 and GLP‐2 have distinct roles:

- GLP‐1: Primarily involved in glucose regulation. It enhances insulin secretion, suppresses glucagon secretion (a hormone that increases blood sugar levels), delays stomach emptying, and promotes feelings of satiety or fullness.^13^
- GLP‐2: Primarily involved in the maintenance and repair of the intestinal lining. It has been shown to improve nutrient absorption, reduce gut permeability, and stimulate cell proliferation in the gut.^14^

So, while they are derived from the same gene, share a similar name, and are co‐secreted, they regulate different stages of digestion within the human body. The researchers also discovered that the glucagon gene could encode multiple peptides, including GLP‐1,^15^ with varying physiological effects.

#### 1.2.1 Glucagon‐like peptide‐1 (GLP‐1) and mimetics

GLP‐1 was found to have multiple effects that make it an attractive target for treating diabetes. These include the stimulation of insulin secretion, the inhibition of glucagon secretion (thus decreasing hepatic glucose output), the reduction of gastric emptying, and the promotion of satiety.^16^ On a molecular level, GLP‐1 is a gastrointestinal peptide hormone composed of 30 amino acids (H‐His‐Ala‐Glu‐Gly‐Thr‐Phe‐Thr‐Ser‐Asp‐Val‐Ser‐Ser‐Tyr‐Leu‐Glu‐Gly‐Gln‐Ala‐Ala‐Lys‐Glu‐Phe‐Ile‐Ala‐Trp‐Leu‐Val‐Lys‐Gly‐Arg‐Gly‐OH) that is predominantly synthesized by the post‐translational modification of proglucagon.^17^, ^18^, ^19^, ^20^

**FIGURE 2:**
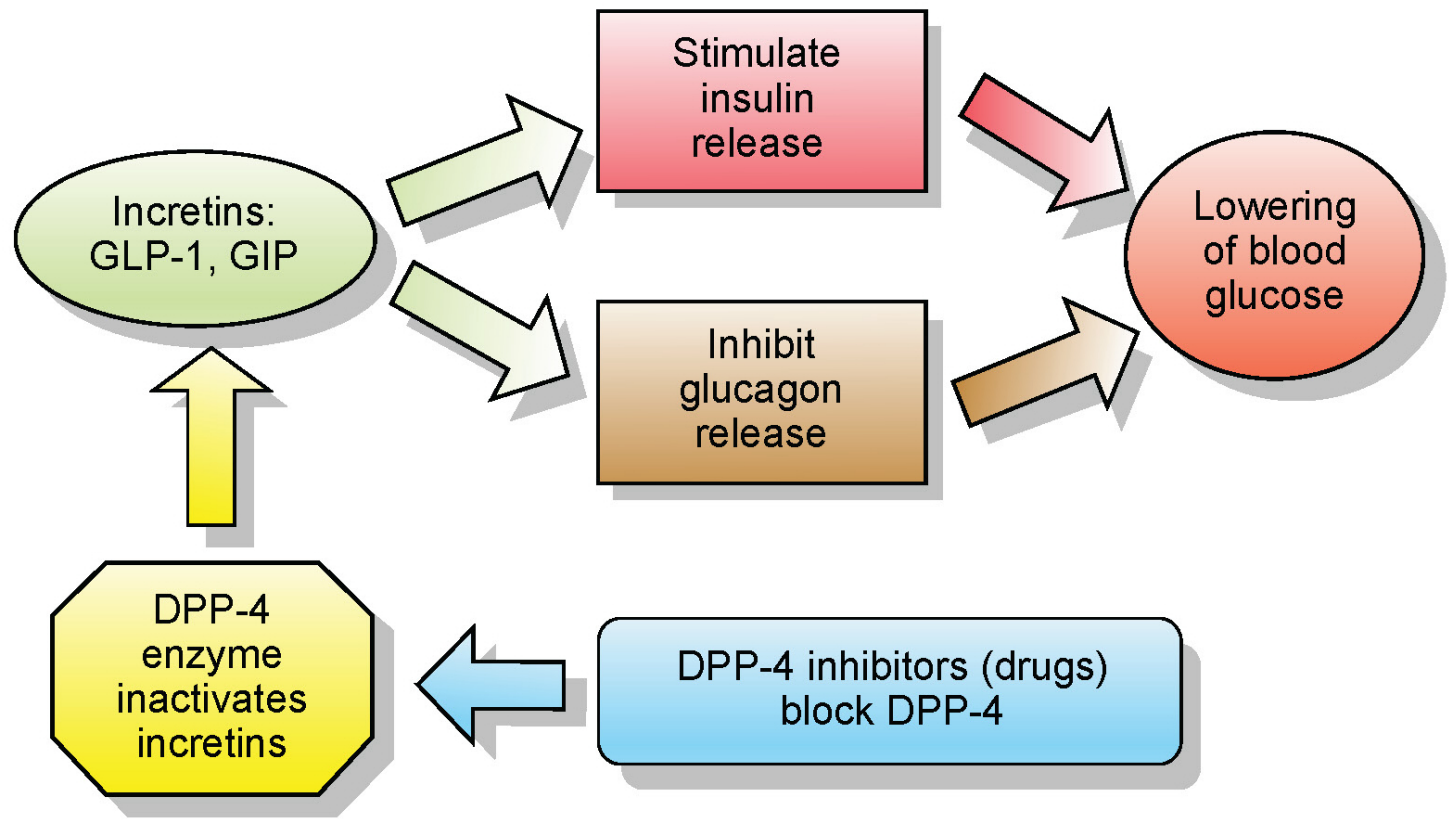
GLP‐1 and diabetes. [By Clinical Cases, Ilmari Karonen ‐ Drawn in Inkscape by Ilmari Karonen based on w:Image:Incretins and DPP 4 inhibitors.jpg from http://casesblog.blogspot.com/2006/11/dpp‐4‐inhibitors‐for‐treatment‐of.html (uploaded by author), CC BY‐SA 3.0, https://commons.wikimedia.org/w/index.php?curid=1540751].

GLP‐1 is a PTM product produced by convertase PC1/3, which releases the equipotent peptides GLP‐1 (7–36 amide) and GLP‐1 (7–37) after cleaving pre‐proglucagon.^21^, ^22^ It stimulates insulin release, enhances insulin sensitivity in peripheral tissues, and slows down the stomach's rate of emptying its contents into the small intestine. Due to the abovementioned abilities, it has therapeutic promise outside of type 2 diabetes. It helps control appetite and promotes a feeling of fullness, aiding in weight management and making it a proper target in drug discovery. The most significant effects of GLP‐1 involve regulating blood glucose levels in individuals with type 2 diabetes by promoting glucose‐dependent insulin release, inhibiting glucagon production, stimulating the growth of beta cells, enhancing insulin secretion, and curbing weight gain. Nevertheless, the relatively brief duration of its activity (approximately 2 min) and its susceptibility to degradation by the enzyme dipeptidyl peptidase‐4 (DPP‐4) restrict the practicality of using GLP1 for therapeutic purposes.^23^ (Figure 2).

Being a potent insulin secretagogue, it has a short half‐life in the body due to rapid cleavage by the widely expressed enzyme DPP‐4 (dipeptidyl peptidase‐4). This cleavage gives rise to GLP‐1 (9–36 amide) and GLP‐1 (9–37) fragments, which are unable to activate the GLP‐1 receptor (GLP‐1R) and, consequently, lose their insulin‐stimulating ability. Despite this limitation, GLP‐1 may still exert GLP‐1R‐independent effects. To overcome the challenges posed by the rapid degradation of GLP‐1, researchers developed GLP‐1 receptor agonists. These compounds mimic the actions of GLP‐1 but are engineered to resist DPP‐4 degradation.^24^

As a result, GLP‐1 receptor agonists have a prolonged half‐life in the body, making them suitable therapeutic agents for managing diabetes and related metabolic disorders. Their extended duration of action allows for sustained activation of the GLP‐1 receptor and offers promising clinical benefits in terms of glycemic control and other physiological effects.

**FIGURE 3:**
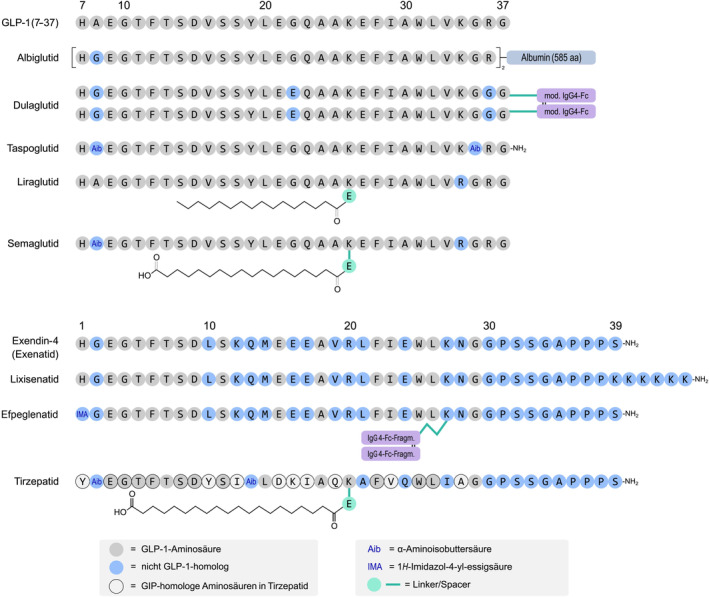
GLP‐1 Sequences [Benff, CC BY‐SA 4.0 <https://creativecommons.org/licenses/by‐sa/4.0>, via Wikimedia Commons].

One of the key differences between GLP‐1 agonists and the natural GLP‐1 hormone is the presence of specific chemical substitutions or additions to the peptide structure of the agonists. These modifications improve their pharmacokinetic properties, allowing them to be administered as injections with a longer half‐life, typically once a week or once a month, compared to the natural GLP‐1 hormone, which has a very short half‐life in the bloodstream. Moreover, the exact structure of specific GLP‐1 agonists can vary depending on the formulation and brand. These agents may have different amino acid sequences and structural characteristics. Despite the differences in their structures, both GLP‐1 agonists and the natural GLP‐1 hormone interact with the GLP‐1 receptors in the body to elicit similar effects, such as stimulating insulin secretion, inhibiting glucagon release, delaying gastric emptying, and promoting a sense of fullness, which can lead to better glucose control and weight management in people with type 2 diabetes (Figure 3).

Exenatide was the first GLP‐1 receptor agonist approved by the FDA in 2005. Exenatide (Byetta, Bydureon) is a synthetically modified version of a naturally occurring hormone, exendin‐4, found in the saliva of the Gila monster, a lizard native to the southwestern United States.^25^ Other approved GLP‐1 agonists include: exenatide; liraglutide (Victoza); dulaglutide (Trulicity); semaglutide (Ozempic, Rybelsus); lixisenatide (Adlyxin); and albiglutide (Tanzeum, Eperzan). The development and approval of GLP‐1 receptor agonists have significantly impacted the treatment of type 2 diabetes, offering another treatment option for patients not adequately controlled by other therapies.

It is worth noting that the GLP‐1 does not compete with glucagon for receptor binding. GLP‐1 and glucagon bind to different receptors and affect glucose metabolism differently. GLP‐1 binds to the GLP1R, primarily on beta cells in the pancreas, while glucagon binds to GCGR in the liver, brain, adipocytes, heart, and kidney.^13^ Despite their opposing roles, GLP‐1 and glucagon work together to maintain glucose homeostasis. While glucagon increases blood glucose levels when they are too low, GLP‐1 reduces blood glucose levels when they are too high, mainly by stimulating insulin secretion and inhibiting glucagon secretion.^16^

#### 1.2.2 Glucagon‐like peptide‐2

In contrast to GLP‐1 and glucagon, glucagon‐like peptide‐2 (GLP‐2)^26^ is a 33 amino acid long hormone (H‐His‐Ala‐Asp‐Gly‐Ile‐Phe‐Thr‐Asp‐Ser‐Tyr‐Ser‐Lys‐Tyr‐Leu‐Asp‐Ser‐Ile‐Gly‐Tyr‐Gly‐Trp‐Leu‐Asn‐Thr‐Gly‐Ser‐Thr‐Gly‐OH) that promotes the intestinal growth. This hormone plays an important role in maintaining the health and integrity of the gut lining and stimulating the absorption of nutrients from food.^27^ Although GLP‐2 shares its name and origin with GLP‐1, its effects on insulin secretion are comparable to, but less dramatic than, those of GLP‐1 on stomach motility and acid production. In fact, both the hormones' agonists are used to treat completely different diseases. GLP‐2 functions primarily on the intestines, stimulating intestinal cell growth and proliferation, increasing blood flow to the gut, and enhancing nutrient absorption. It plays a vital role in maintaining the intestinal mucosal lining and promotes recovery after gut injury.

Like other peptide hormones, GLP‐2 is rapidly degraded by enzymes in the body, so synthetic analogues have been developed to extend its half‐life and improve the therapeutic potential for conditions involving intestinal dysfunction and malabsorption. Synthetic analogues of GLP‐2 have been developed to enhance its stability and duration of action. One such analogue is teduglutide, which is used to treat short bowel syndrome (SBS) in adults. Teduglutide works by mimicking the effects of GLP‐2, thereby helping to improve the absorption of nutrients.^28^

Given the therapeutic potential of GLP‐1, GLP‐2 and glucagon, researchers have developed their analogues. However, creating GLP‐1 receptor binding chemicals (GLP‐1R agonists), including exendin‐4 analogues and DPP‐4 inhibitors, to enhance the stability and activity of endogenous GLP‐1 or prevent its degradation, is at boom. Some of these GLP‐1R agonists are already in clinical use, with many more currently being developed, and are likely to enhance their ease of administration, tolerability, and effectiveness. Studies have revealed that although the GLP1R agonists, like liraglutide/semaglutide, and analogues, like exenatide, have a direct effect on the production of insulin from pancreases, they also have pleiotropic effects on various organs and systems in the body, including the brain, stomach, and heart—with no evident effect on the liver system majorly due to the absence of L cells.^29^

### 1.3 GLPR and GCGR agonists

**FIGURE 4:**
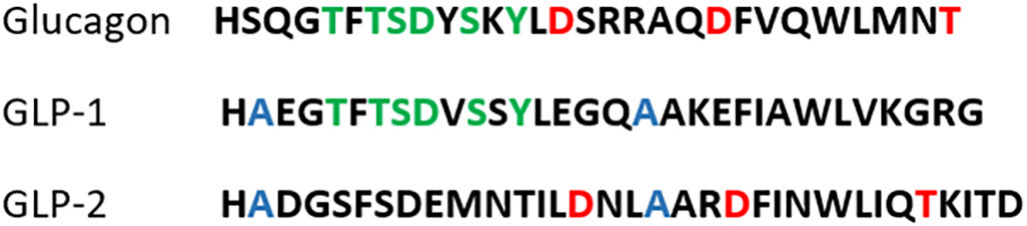
GLP‐2 and its analogue teduglutides sequence alignment to present sequence similarity.

The glucagon‐like peptide‐1 receptor (GLP1R), glucagon‐like peptide‐2 receptor (GLP2R), and glucagon receptor (GCGR) all collectively play essential roles in regulating glucose homeostasis and intestinal function. The multiple sequence alignment (MSA) and phylogenetic tree analysis of glucagon‐like‐peptide‐1 receptor agonists (GLP1RAs), including semaglutide, lixisenatide, dulaglutide, tirzepatide, exenatide, liraglutide, ecnoglutide and efpeglenatide, reveal the close evolutionary relationships between these agents (Figure 1). The phylogenetic values provided for the GLP1RAs in Figure 1 represent the branch lengths in the phylogenetic tree constructed using multiple sequence alignment (MSA) of GLP1RAs amino acid sequences. Branch lengths in a phylogenetic tree represent genetic distances or evolutionary differences between sequences. Longer branches indicate greater genetic divergence or evolutionary time, while shorter branches suggest closer evolutionary relationships. The negative phylogenetic values (shorter branches) for ecnoglutide, dulaglutide, semaglutide and lixisenatide, indicate that they are closely related regarding their sequence similarity. The shorter branches indicate that these sequences have relatively low genetic divergence or evolutionary differences, and they may share a more recent common ancestor. In contrast to this, based on the sequences, the positive phylogenetic values (longer branches) for dulaglutide, tirzepatide, exenatide, liraglutide and efpeglenatide, indicate that they are more distantly related in terms of sequence similarity. The longer branches imply that these sequences have greater genetic divergence or evolutionary differences and may have diverged from their common ancestors. A similar sequence comparison for the GLP‐2 and its mimetic teduglutide is shown in Figure 4.

Despite the differences in phylogenetic values and slight variations in the amino acid sequences, these GLPRAs target the same receptor. Through their interaction with the GLP‐1 receptor, these GLPR1A drugs elicit similar effects to endogenous GLP‐1, leading to improved glucose control, increased insulin secretion, reduced glucagon release, and decreased appetite. Although sequence conservation and homogeneity underpin their similar mechanisms of action, the slight variations in their amino acid sequences or chemical substitutions contribute to differences in their pharmacokinetics, durations of action and interactions with the receptor, resulting in varying pharmacokinetic profiles and clinical effects (Table 1).

**TABLE 1:** Pharmacokinetic profiles of a few GLP‐1 receptor agonists.

Drug	Administration	*t*_1/2_	*T*_max_	Molecular Weight
Semgalutide	Once weekly	7 days	1.5 h	4.11
Liraglutide	Once daily	11–15 h	9–12 h	3.75
Dulaglutide	Once weekly	5 days	48 h	59.67
Exenatide ER	Once weekly	2.4 h	2 weeks	4.12
Lixisenatide	Once daily	2.8 h	1.25 h	4.86

These variances in their pharmacokinetic profiles are also evident in the cyclic way they are administered daily or weekly. For instance, liraglutide has a longer half‐life than semaglutide because it is post‐translationally modified to have an albumin‐binding moiety, allowing it to bind to albumin in the blood. This binding slows down the elimination of liraglutide from the body, leading to a longer duration of action and a half‐life of approximately 13 h. On the other hand, semaglutide is designed with a fatty acid side chain that contributes to a slower absorption rate and clearance from the body. This modification results in a longer half‐life for semaglutide, approximately 7 days; hence, the extended half‐life allows for once‐weekly dosing, as demonstrated in Table 1. As an engineered GLP‐1 agonist, Exenatide is resistant to degradation by DPP‐4 enzymes, resulting in an extended half‐life in the bloodstream. This allows for convenient administration as either a twice‐daily or once‐weekly injection. Although it has a relatively shorter duration of action than other GLP‐1 agonists, exenatide offers flexibility in dosing frequency, which may be advantageous for some patients.

Similarly, dulaglutide, designed as a once‐weekly GLP‐1 agonist, has a larger molecular size due to its fusion with an immunoglobulin G (IgG) molecule. This modification enhances stability and extends the duration of action, allowing for a once‐weekly dosing regimen. Due to its enormous molecular weight, it has a relatively higher *T*_max_ than other GLP1RAs. *T*_max_ governs the rate of drug absorption and the rate of drug elimination.

Overall, while all these GLP‐1 agonists act through the GLP‐1 receptor to elicit beneficial effects on glucose metabolism, their distinct structural and, hence, distinctive pharmacokinetic properties allow for variations in dosing regimens and durations of action. Liraglutide's daily sustained effect, semaglutide's extended duration of action, exenatide's flexibility in dosing frequency and dulaglutide's once‐weekly dosing provide physicians with options to tailor treatment based on individual patient needs and preferences.

The variations in their pharmacokinetics highlight the importance of selecting the most appropriate GLP‐1 agonist to optimize patient outcomes in managing diabetes and related metabolic disorders. In contrast to both GLP1R and GLP2R agonists, as discussed previously, the mimetics attached to the GCGR have a different mode of action. These GCGR mimetics activate the glucagon receptor and increase glucagon signalling. Unlike GLP1R agonists, which reduce glucagon release, GCGR mimetics aim to stimulate glucagon secretion. They have potential applications in treating various metabolic disorders, including diabetes and obesity. However, their development is more challenging due to the risk of inducing hyperglycemia and increased hepatic glucose production. Since the GCGR agonists have emerged as potential therapeutic options for managing obesity, diabetes and other metabolic disorders, one intriguing development in this field is the discovery of unimolecular dual agonists that simultaneously activate both the GLP1R and the GCGR.

These dual agonists, such as oxyntomodulin (OXM) and DA‐1726, have demonstrated promising effects on body weight reduction and blood glucose control, highlighting their potential as innovative treatments.^30^ Interestingly, research has also delved into dual incretin receptor agonists, like JNJ‐64565111 and cotadutide, which have shown superior efficacy in inducing weight loss compared to single agonists.^31^, ^32^ Additionally, numerous multi‐agonists capable of co‐agonism between GLP‐1 and glucagon receptors, such as BI 456906, LY3305677, LY4347943, JNJ‐54729518, HM15211, NNC9204‐1706, Alt‐801 and G3215, are currently in development. These multi‐agonists hold great promise in achieving enhanced therapeutic outcomes through dual activation of both receptors.^33^, ^34^ In conclusion, developing GCGR agonists, particularly unimolecular dual and multi‐agonists, represents a promising avenue for addressing obesity, diabetes and metabolic disorders. While these findings are encouraging, further research is essential to optimize these agonists' potency, balance and safety to maximize their efficacy and minimize potential adverse effects.

## 2 CLINICAL TRIALS

The approval of the first semaglutide drug on the 5 December 2017 marked a significant milestone, paving the way for an explosion of research and development in the field of GLP1R agonists (GLP1RAs); around 1007 studies have been conducted according to the clinicaltrial.org data,^35^ and numerous agonists have hit the market till 2023. In the last few years, the GLP1RA class has expanded drastically, and numerous agents are now available for treating various disease conditions globally. Each drug may have different benefits and drawbacks due to the differences in their effectiveness and tolerability, dose frequency, administration needs, and cost across the medicines in the same class. Clinical GLP1RA investigations have demonstrated on multiple fronts that all the GLP1RAs are potent HbA1c‐lowering agents. However, there are variations in the strength of the impact on HbA1c, physiological aspects, and the frequency of negative effects. Of these 1007 clinical studies, only 414 were completed successfully, with 180 focused majorly on conditions like pre‐diabetes, diabetes mellitus (type 1 and 2), latent autoimmune diabetes, cystic fibrosis‐related diabetes, gestational diabetes, etc. The remaining 234 clinical studies focused on other conditions like hypoglycemia, metabolic and bowel conditions, obesity, chronic kidney disease, etc.

**FIGURE 5:**
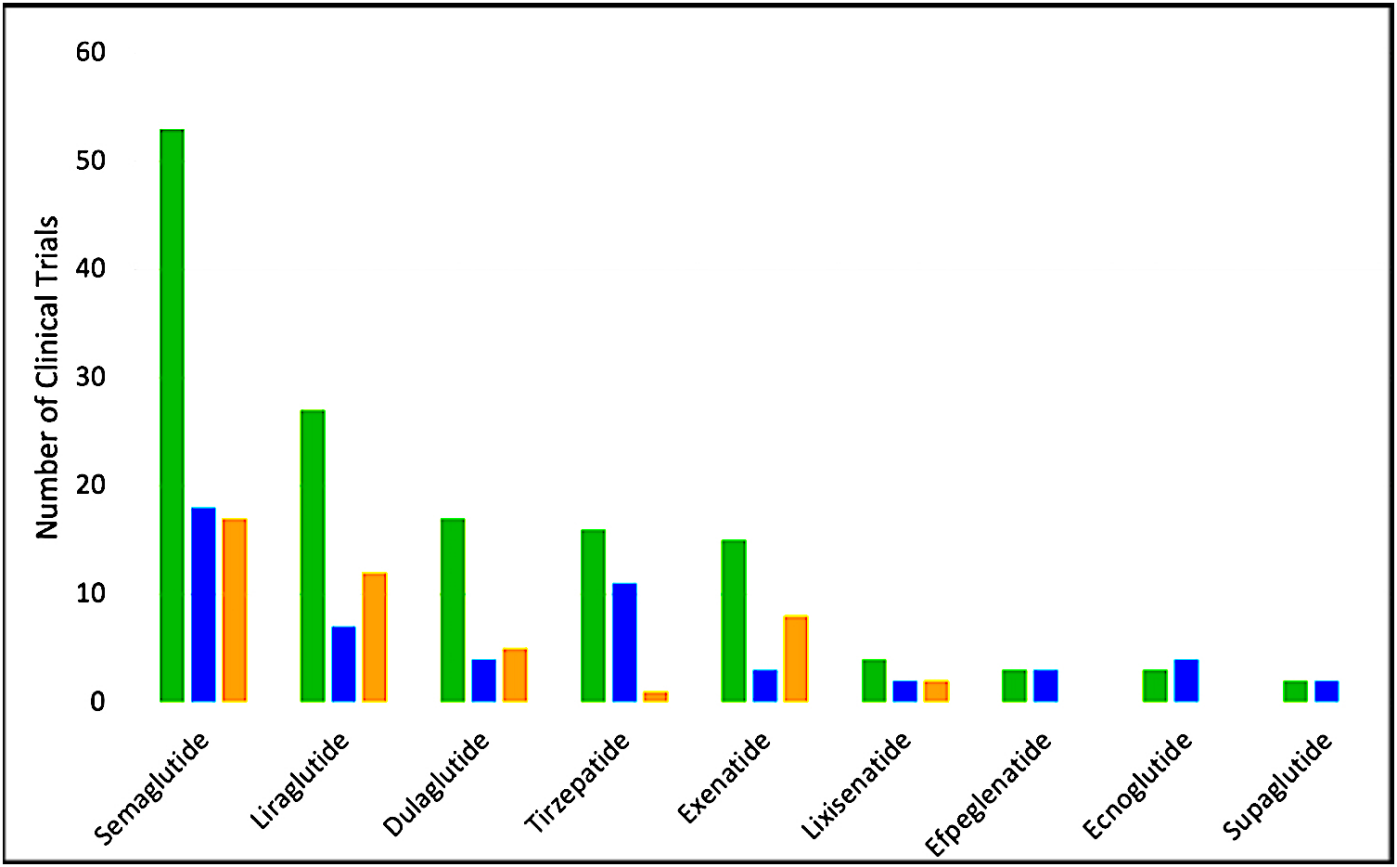
Distribution of Clinical Studies on GLP1RAs across Phases 1 to 3 (left to right).

Three hundred sixty‐nine interventional studies^36^ are currently in the clinical phase, with 101 in phase 3 and 87 in phase 4. Semaglutide has long been a pivotal therapeutic, and since 2017, around 53 studies (phases 1–4) have been conducted on semaglutide alone. Besides semaglutide, GLP1R analogues like liraglutide, tirzepatide, exenatide, lixisenatide, dulaglutide, efpeglenatide, ecnoglutide and eupaglutide are now also being prioritized by developers with multiple studies being in phase 1–3 as shown in Figure 5.

All these GLP1RAs bind and activate the GLP1 receptor similarly to the endogenously produced GLP1; hence, they have similar homology and sequence identity with slight variations.^21^ Long‐acting GLP1RAs, such as liraglutide and once‐weekly exenatide, dulaglutide, albiglutide and semaglutide, have more profound effects on overnight and fasting plasma. Short‐acting GLP‐1 RAs, such as lixisenatide, have short‐lived peaks in plasma drug concentrations following each injection, with intermittent periods of near‐zero concentrations. Tirzepatide, on the other hand, is a dual GIP/GLP‐1 receptor co‐agonist that is more effective in reducing HbA1c and body weight than semaglutide. Multiple clinical studies have been conducted on these agents to analyse their therapeutic potential and pharmacological aspects; some of each is presented below in relative comparison to the semaglutide (Table 2).

### 2.1 Liraglutide

Liraglutide and semaglutide are both long‐acting GLP‐1RAs that have pharmacodynamic effects 24 h a day. They have around 93% sequence similarity with slight sequential and structural variances; therefore, they act similarly (Figure 4).^36^ Although their administration intervals and doses may vary, both are well‐tolerated and effective for weight loss.^37^ A systematic review of 18 studies reported that liraglutide and semaglutide therapies led to a weight loss of 48.2%–88.7% among obese and overweight adults without diabetes, where overall clinically acceptable change was set to be greater than 5%.^38^ Furthermore, a phase 2 trial found that once‐daily administration of subcutaneous semaglutide 0.4 mg dose (equivalent to 2.8 mg once weekly) significantly increased weight loss compared to the liraglutide 3.0 mg dose.^39^ Another randomized clinical trial compared the efficacy and adverse effects profiles of once‐weekly subcutaneously administered semaglutide, 2.4 mg, versus once‐daily liraglutide, more than 1.8 mg. The study backed the previous findings that both medications were significantly associated with weight loss. However, semaglutide induced greater weight loss and had fewer permanent treatment discontinuations due to adverse events.

In contrast, another study testing the weight‐loss effects of semaglutide presented those 85 patients (48.6%) reported experiencing adverse effects associated with gastrointestinal symptoms being the most commonly reported ones.^40^ Based on data from 10 studies involving liraglutide and semaglutide, both GLP‐1 receptor agonists demonstrated a generally favourable safety profile in real‐world practice. The discontinuation rates due to adverse events were relatively low, with liraglutide 3.0 mg once‐daily groups ranging from 5.4% to 11.4% and semaglutide 2.4 mg per week groups ranging from 2.4% to 7.0%. The overall incidence of adverse events was higher in the liraglutide and semaglutide groups (ranging from 66.5% to 96.7% and 81.3% to 95.8%, respectively) than in the control groups. However, it is important to note that serious adverse events were infrequently reported, ranging from 0% to 7.5% in liraglutide groups and 7.7% to 9.8% in semaglutide groups, with most being mild to moderate in severity.

A STEP 1 trial showed that the total weight loss from the liraglutide therapy was around 5.4%, with an estimated cost of the entire trial being $17,585. Similarly, in a SCALE study using semaglutide, 12.4% weight loss was observed, with an estimated trial cost of $22,878. This resulted in a conclusion that the cost per 1% of weight for semaglutide was $1845, significantly lower, and hence more efficient than liraglutide, which was $3256.^41^

### 2.2 Exenatide

Exenatide and semaglutide are similar in homology, with 42.5% sequence identity and 52.5% sequence similarity. They have significantly improved glycemic control, reducing HbA1c levels in clinical trials. Semaglutide has shown superior HbA1c reduction compared to exenatide in some head‐to‐head studies, and both have shown cardiovascular benefits in specific clinical trials, with reductions in the risk of major adverse cardiovascular events (MACE) reported.

Phase 3a, open‐label, parallel‐group, randomized controlled trial, SUSTAIN 3^42^ study conducted in 2013 evaluated the efficacy and safety of semaglutide once‐weekly versus exenatide extended‐release (ER). The study comprised 813 subjects and showed that after 56 weeks of treatment, Semaglutide 1.0 mg outperformed exenatide ER 2.0 mg in improving glycemic control and reducing body weight. Despite these differences in efficacy, both drugs had similar safety profiles. These findings suggest semaglutide is a highly effective treatment option for individuals with T2D who have inadequate control of oral antidiabetic medications.

A randomized, double‐blind, placebo‐controlled trial found that exenatide positively affected off‐medication motor scores in Parkinson's disease (PD) patients, and these effects were sustained beyond the treatment period.^43^ A review stated that there is uncertainty regarding whether exenatide may improve motor symptoms in PD; this conclusion was based on limited evidence from small studies, and hence, there are clinical trials currently to prove any correlation between exenatide and PD.^44^, ^45^ A proof‐of‐concept trial was also conducted in PD patients to investigate the potential beneficial effects of exenatide on the survival of dopamine cells responsible for the secretion of dopamine, a ‘happy hormone’. The preliminary findings showed improvements in motor and non‐motor symptoms compared to the placebo group. There are also ongoing trials to test the effects of exenatide on the release of oxytocin, another happy hormone responsible for appetite, psychopathology, prosocial behaviour, and sexual function.^46^ Another multicenter, double‐blind, randomized, placebo‐controlled phase 3 trial called the ‘Exenatide‐PD3’ study aimed to evaluate exenatide as a potential disease‐modifying treatment for PD.^47^, ^48^ Previous phase 2 clinical studies have shown a delay in the progression of motor symptoms in PD patients treated with exenatide. Furthermore, a study revealed that combination therapy of metformin and exenatide was more effective than metformin alone in overweight/obese women with PCOS. The combination treatment resulted in significant reductions in body weight, BMI, and waist circumference while also showing improvements in insulin sensitivity.^49^, ^50^ These findings have generated interest and support for further research.

### 2.3 Dulaglutide

Dulaglutide and semaglutide are dosed once weekly and used to treat T2D. They both reduce blood glucose by stimulating insulin secretion and reducing glucagon secretion, both occurring in a glucose‐dependent manner. Regarding warnings and precautions, dulaglutide does not include the precaution of retinopathy, while the incidence of diabetic retinopathy was reported in the SUSTAIN 6 trial for semaglutide.^41^ An indirect treatment comparison study showed that semaglutide 1.0 mg was significantly more effective than dulaglutide 3.0 mg and comparable with dulaglutide 4.5 mg in reducing body weight from baseline.^51^ Another study found that semaglutide is superior to dulaglutide for weight loss and improving glycemic control in patients with type 2 diabetes.^52^ A clinical trial reported that semaglutide was more potent and effective in weight reduction than dulaglutide. Another study found that semaglutide 2.0 mg was significantly more effective at reducing body weight from baseline than dulaglutide 3.0 and 4.5 mg.^53^ Another study investigated the potential effect of dulaglutide on women with diabetes and polycystic ovaries (PCOS).^54^ This study concluded that although dulaglutide did not produce better results than the calorie‐restricted diet, it increased weight loss and improved HbA1c levels in women with PCOS (Table 2).

**TABLE 2:** Comparison of GLP‐1 receptor agonists: liraglutide, exenatide, dulaglutide, and tirzepatide.

Feature	Liraglutide	Semaglutide	Exenatide	Dulaglutide	Tirzepatide
Type of drug	GLP‐1 receptor agonist	GLP‐1 receptor agonist	GLP‐1 receptor agonist	GLP‐1 receptor agonist	Dual GIP and GLP‐1 receptor agonist
Frequency of administration	Once daily	Once weekly	Twice daily or once weekly	Once weekly	Once weekly
Efficacy for glycaemic control	Good	Excellent	Good	Good	Excellent
Efficacy for weight loss	Good	Excellent	Good	Good	Excellent
Reduces risk of major adverse cardiovascular events	No	Yes	No	No	No
Other indications	Obesity	Obesity, non‐alcoholic steatohepatitis, Wolfram syndrome	Parkinson's disease, non‐alcoholic fatty liver disease, Wolfram syndrome	Polycystic ovary syndrome (PCOS)	PCOS, Short bowel syndrome, Wolfram syndrome

The effects of dulaglutide on the regulation of gonadal hormones were tested clinically as well. Still, they resulted in no clinically significant effect on sexual desire or analysed hormones of the gonad axis in men.^55^ Furthermore, ongoing trials aim to test the impact of dulaglutide‐related insulin secretion in adults with insufficient pancreatic cystic fibrosis and abnormal glucose tolerance.^56^ Other trials aim to test the effects of dulaglutide on metabolism and ectopic fat deposition in chronic kidney disease.^57^

### 2.4 Tirzepatide

Tirzepatide and semaglutide are both potent GLP‐1 receptor agonists used to treat T2D. Like semaglutide, tirzepatide offers the convenience of a once‐weekly subcutaneous injection but has the unique advantage of being a dual GIP (gastric inhibitory polypeptide) and GLP1RA. This dual action allows tirzepatide to concurrently target multiple pathways involved in glucose regulation, potentially providing additional benefits in managing diabetes. Both medications have shown significant efficacy in improving glycaemic control and are generally well‐tolerated, but tirzepatide's distinct pharmacological profile holds promise in enhancing diabetes management outcomes. A 40‐week, phase 3 trial, randomized trial compared the efficacy and safety of tirzepatide and semaglutide in patients with T2D.^58^ The study found that tirzepatide was superior to semaglutide regarding the mean change in glycated haemoglobin level. Another study compared the effects of tirzepatide 15 mg to placebo and semaglutide on additional factors such as α and β cell function and insulin sensitivity. A study found that tirzepatide demonstrated impressive glycaemic efficacy in T2D by enhancing critical aspects of diabetes pathophysiology.^59^, ^60^ Notably, it improves β‐cell function, insulin sensitivity, and glucagon secretion.

These substantial improvements contribute to the remarkable glucose‐lowering capabilities of tirzepatide. A systematic review and meta‐analysis of the efficacy and safety of tirzepatide in patients with T2D found that tirzepatide decreased HbA1c by 0.36% and fasting serum glucose by 13.00 mg/dL when compared with GLP‐1 RAs (semaglutide 1 mg and others).^61^ Another study found that tirzepatide significantly reduced HbA1c and body weight more effectively than semaglutide.^62^ While tirzepatide is primarily studied for its efficacy in treating T2D and obesity, ongoing clinical trials are investigating its potential use in other conditions, such as Wolfram syndrome—an orphan disease. Currently, trials are also ongoing to test the effects of non‐alcoholic steatohepatitis in the United Arab Emirates population. The main aim of this trial is to provide insight into the impact on fat deposition in the liver after 6 months of treatment since non‐alcoholic fatty liver disease is a significant problem in T2D and obesity patients.^63^

## 3 DURATION OF ACTION OF GLP1RAS

With the approval of numerous GLP1RAs, it is necessary to compare, analyse, and determine their suitability for patients. Various meta‐analysis studies have concluded that once‐weekly (i.e. semaglutide) GLP1RA are more effective in reducing HbA1c levels and equally effective in weight loss than twice‐daily injection (i.e. exenatide). However, it was found that they were not superior to liraglutide, a once‐daily injection, regarding the HbA1c reduction. Additionally, a separate study provided further support, showing that long‐acting GLP1RA such as dulaglutide, liraglutide and once‐weekly exenatide were more effective than the twice‐daily exenatide and lixisenatide in lowering fasting plasma glucose levels and HbA1c levels of patients (Table 1). These findings highlight the varying effectiveness of different GLP‐1 receptor agonists in managing glycaemic control and weight loss in patients with type 2 diabetes. The pharmacokinetic/pharmacodynamic profile of each GLP1RA may also vary along with its activity, depending upon its mode of action.

Semaglutide demonstrates high binding affinity to the GLP‐1 receptor, contributing to its potent therapeutic effect. In vitro studies have shown that semaglutide has a binding affinity several‐fold higher than endogenous GLP‐1, enabling it to activate GLP‐1 receptors effectively. The high binding affinity of semaglutide also translates into a long duration of action due to its slow release from the subcutaneous depot. Furthermore, its resistance to degradation by DPP‐4 enzymes contributes to its prolonged receptor occupancy and sustained pharmacological effect. This extended duration of action allows for once‐weekly dosing, providing convenience and improved patient adherence compared to daily or multiple daily dosing regimens. Studies comparing semaglutide with other GLP1RA have demonstrated its superior receptor binding affinity and longer duration of action on multiple instances. These pharmacological properties contribute to the sustained glycemic control and weight management observed with semaglutide treatment.

In contrast to semaglutide, liraglutide's unique structure, incorporating a palmitate side chain at position 26 using a γ‐glutamic acid spacer that sets it apart kinetically from the related compounds, such as exenatide, etc.^64^ This modification allows liraglutide to bind strongly to albumin, resulting in 99% albumin binding compared to natural GLP‐1. As a result, liraglutide can evade glomerular filtration, leading to an extended duration of action. Metabolites of liraglutide are detected in both urine and faeces, and it undergoes hepatic metabolism, being eliminated primarily through the liver and kidneys. Due to its pharmacokinetic profile, liraglutide is considered a desirable treatment option for T2D.^65^

Studies on healthy male subjects receiving multiple subcutaneous doses of liraglutide reported a time to maximum plasma concentration, *T*_max_ of 9–14 h, and a plasma half‐life, *t*_1/2_ of elimination ranging from 6 to 17.9 h, depending on the dosing regimen.^66^ These characteristics make liraglutide a once‐daily agent that offers sustained glucose‐lowering effects, making it suitable for managing T2D effectively and potentially improving patient treatment adherence. When comparing liraglutide with exenatide, several differences may favour using liraglutide. A direct comparison was made in the LEAD‐6 trial, where each agonist's efficacy and safety profiles were assessed. Over 26 weeks, subjects received either 1.8 mg/day of liraglutide (202 subjects) or 10 μg twice daily of exenatide (187 subjects).^67^ This study revealed that liraglutide maintained steady‐state plasma levels for up to 24 h after administration. In comparison, exenatide peaked and returned to baseline plasma levels within 10–12 h following administration, making liraglutide effective for longer.^63^, ^68^, ^69^

Another crucial distinction of liraglutide is its impact on renal function. Liraglutide's chemical structure allows for minimal impact on renal function, whereas exenatide is primarily eliminated through the kidneys.^70^ Consequently, exenatide is not recommended in individuals with severe renal impairment or end‐stage renal disease. Hence, these findings suggest that liraglutide may have advantages over exenatide regarding its pharmacokinetic profile and renal safety, making it a potentially more favourable treatment option for a specific patient population.^40^, ^71^

Like semaglutide, liraglutide, and exenatide ‐ dulaglutide has also shown significant benefits in controlling diabetes. One of the considerable advantages of dulaglutide is its convenient once‐weekly dosing regimen compared to the once‐daily dosing required in the case of liraglutide. Dulaglutide's single dose has demonstrated the ability to lower fasting glucose levels and reduce postprandial glucose concentrations in patients with T2D.^72^ In a study of adults with T2D, once‐weekly dulaglutide treatment resulted in reduced fasting and two‐hour postprandial glucose levels, as well as a decrease in postprandial serum glucose incremental area under the curve, compared to placebo (−25.6 mg/dL, −59.5 mg/dL, and −197 mg/h/dL, respectively).^73^ These effects were sustained after 6 weeks of the 1.5 mg dose.

Additionally, dulaglutide treatment in patients with T2D increased first‐ and second‐phase insulin secretions compared to placebo. Following subcutaneous administration, the pharmacokinetics of dulaglutide become evident. With a median of 48 h, attaining the maximal concentration at a steady state ranges from 24 to 72 h. After once‐weekly treatment, steady‐state concentrations are usually reached in 2–4 weeks. The average peak plasma concentration and total systemic exposure (area under the curve) of dulaglutide were determined to be 114 ng/mL and 14,000 ng·h/mL, respectively, after multiple‐dose administration of 1.5 mg to a steady state. The accumulation ratio was approximately 1.56, indicating minimal drug accumulation over time. Importantly, there was no statistically significant difference in exposure levels based on the administration sites.

Furthermore, the mean absolute bioavailability of single 0.75‐ and 1.5‐mg doses of dulaglutide was determined to be 65% and 47%, respectively. Additionally, the mean volumes of distribution were found to be approximately 19.2 and 17.4 L, respectively. These pharmacokinetic parameters provide valuable insights into the characteristics of dulaglutide after subcutaneous administration and contribute to its once‐weekly dosing convenience in managing T2D.

Dulaglutide is assumed to undergo degradation into its constituent amino acids upon administration. However, at steady state, the mean apparent clearance for the 0.75‐mg dose is approximately 0.111 L per hour, and for the 1.5‐mg dose, it is around 0.107 L per hour. The elimination half‐life (*t*_1/2_) of dulaglutide for both doses is approximately 5 days. Despite belonging to a class of medications requiring caution and renal and hepatic function monitoring, dulaglutide does not necessitate dose adjustments. Its pharmacokinetic profile and prolonged half‐life (*t*_1/2_) support its once‐weekly dosing convenience and provide valuable information for its safe and effective use in managing T2D.^74^

In contrast to dulaglutide, another GLP1RA, lixisenatide, demonstrates a pharmacokinetic response dependent on the administered dose.^75^ In a 4‐week, randomized, and placebo‐controlled study involving 64 patients with T2D, once‐daily doses of lixisenatide were administered in the 5–20 μg range, with increments of 2.5 μg every fifth day.^76^ The study observed a proportional increase in steady‐state plasma concentrations with the dose administered (5, 10, and 20 μg). The mean area under the curve (AUC) and peak plasma concentration also showed a dose‐dependent increase, correlating with the dose and dosing frequency. Peak plasma concentrations were achieved within 1.25–2.25 h, depending on the specific dose.^77^, ^78^ Importantly, all doses of lixisenatide (5–20 μg) were associated with reduced AUC for postprandial glucose (PPG) compared to placebo, with the most significant decrease observed with the 20 μg dose of lixisenatide.^79^, ^80^ These findings emphasize the dose‐dependent pharmacokinetic profile of lixisenatide and its effectiveness in lowering postprandial glucose levels.^81^, ^82^

Furthermore, tirzepatide, a dual GIP and GLP‐1 receptor agonist, is a valuable therapeutic option for managing T2D in adults alongside diet and exercise due to its ability to target two receptors. Extensive research on its pharmacokinetics has revealed significant findings. The drug exhibits a slow onset of action, taking approximately 4 weeks to reach steady‐state levels, indicating the need for patience during the initial phase of treatment. Its pharmacokinetics remain consistent between healthy subjects and patients with T2DM, irrespective of food intake and exposure. Another notable attribute of tirzepatide is its low molecular weight of 4.8 kDa, which falls below the glomerular filtration threshold, suggesting that the kidneys are unlikely to eliminate it. Additionally, the drug displays imbalanced agonism at the GIPR and GLP‐1R, leading to biased signalling at the GLP‐1R. This novel mechanism of action underlines the uniqueness of tirzepatide as a treatment option for type II diabetes, making it a promising avenue for improved glycemic control in affected individuals.^83^, ^84^

Besides tirzepatide, the efficacy, safety, and pharmacokinetic/pharmacodynamic characteristics of efpeglenatide, a long‐acting GLP1RA, were tested clinically.^85^ Two studies concluded that efpeglenatide serum concentrations increased proportionally with the administered dose. The median time to reach maximum serum concentration (*t*_max_) ranged from 72 to 144 h in the single‐dose study and from 48 to 120 h in the repeated‐dose study (after the final dose). The geometric mean half‐life (*t*_1/2_) varied from 135 to 180 h across the studies. In the repeated‐dose study, peak‐to‐trough ratios were 1.3 to 1.4 with once‐weekly dosing and 5.9 to 12.9 with once‐monthly dosing. It was observed that the repeated doses of efpeglenatide resulted in significant reductions in glycated haemoglobin compared to the placebo group.^86^, ^87^ The estimated *t*_1/2_ values for efpeglenatide once weekly and once monthly were approximately 6 days and 6–7 days, respectively, and were comparable to the ranges (5–8 days) reported for dulaglutide and semaglutide. Furthermore, the observed delayed *t*_max_, extended half‐life, and low peak‐to‐trough ratios indicate the potential for improved efficacy and dosing flexibility, highlighting efpeglenatide as a well‐tolerated and promising treatment option for patients with T2D.^88^ Besides this, a few other GLP1RAs are under clinical testing to improve patient treatment options. Ecnoglutide^89^ is one of such GLP1RAs. It is subcutaneously administered and is currently under development for treating T2D, obesity, non‐alcoholic steatohepatitis, and liver fibrosis.

The availability of multiple GLP1RAs has expanded treatment options, providing patients with the opportunity to choose the one that best suits their individual needs and preferences. When exploring the landscape of clinically approved and available GLP1RAs and those under testing, it becomes evident that the increased availability of these options offers patients a range of choices for their treatment. Given its established efficacy and safety profile, Semaglutide is a reference point in this comparison.

## 4 CLINICAL APPLICATIONS

Glucagon‐like peptide‐1 (GLP‐1) agonists are medications primarily used to manage type 2 diabetes mellitus (T2DM); however, they have been shown to improve the risk of cardiovascular, neurogenerative, and polycystic ovaries diseases, etc.^90^

### 4.1 Treatment of type 2 diabetes mellitus

In patients with T2DM,^91^ GLP‐1 agonists work by several mechanisms:

- They stimulate insulin secretion from pancreatic beta cells in a glucose‐dependent manner, promoting insulin secretion when glucose levels are elevated.
- They inhibit glucagon secretion, which prevents the liver from producing excess glucose.
- They slow gastric emptying, which can reduce the rate at which glucose enters the bloodstream after eating.
- They increase satiety, leading to a decrease in food intake and potentially promoting weight loss.

### 4.2 Cardiovascular effects

Moreover, several GLP‐1 agonists have been demonstrated to have cardiovascular benefits in large clinical trials. Liraglutide dramatically decreased the risk of severe adverse cardiovascular events in individuals with T2DM and high cardiovascular risk, according to the LEADER study.^92^ Similarly, the SUSTAIN‐6 trial presented that semaglutide significantly reduced the risk of cardiovascular events by many folds.^93^ As a result, GLP‐1 agonists are increasingly recognized for their potential role in managing cardiovascular disease in patients with T2DM.

### 4.3 Weight management

GLP‐1 agonists are also being used for weight management and treating obesity. Specifically, liraglutide (at a higher dose than that used for diabetes) and semaglutide have been approved for treating obesity due to their effects on satiety and food intake.^93^

### 4.4 Pre‐diabetes

GLP‐1 agonists are also being explored as a treatment for pre‐diabetes. A 2018 study found that liraglutide can reduce body weight and improve glucose metabolism in people with obesity and pre‐diabetes.^61^

### 4.5 Non‐alcoholic steatohepatitis (NASH)

Several GLP‐1 agonists are also being investigated for treating non‐alcoholic steatohepatitis (NASH), a liver disease often associated with obesity and type 2 diabetes. Research suggests these drugs may reduce liver fat content, inflammation, and fibrosis in patients with NASH.^94^

### 4.6 Neurodegenerative diseases

Pre‐clinical studies suggest that GLP‐1 agonists might have a potential role in treating neurodegenerative diseases such as Parkinson's disease and Alzheimer's disease due to their neuroprotective properties. Still, more research is needed to confirm these effects in humans.^94^

### 4.7 Polycystic ovary syndrome (PCOS)

Some research has suggested that GLP‐1 agonists may help manage polycystic ovary syndrome (PCOS), a condition associated with insulin resistance. Though more research is needed, these drugs may improve menstrual regularity and decrease body weight.^95^

### 4.8 Gastroparesis

GLP‐1 agonists, due to their ability to slow gastric emptying, are also being studied for use in gastroparesis, a condition that can occur in people with diabetes. However, there is an ongoing debate about their effectiveness in this area due to the potential for worsening symptoms, and more research is needed.^96^

### 4.9 Lowering blood pressure

Several GLP‐1 agonists, like liraglutide, have also shown modest effects on lowering blood pressure. It's unclear how this occurs, but it may be related to the weight loss that GLP‐1 agonists can promote.^91^

## 5 CONCLUSION

Glucagon‐like peptide (GLP‐1) receptor agonists (GLP1RAs) have emerged as valuable therapeutic options for managing various diseases. Among these agents, semaglutide is a remarkable once‐weekly GLP1RA with proven clinical efficacy and safety. Furthermore, long‐acting GLP1RAs, such as dulaglutide and liraglutide, have been shown to outperform exenatide twice‐daily and lixisenatide, providing a diverse array of choices for treatment. Clinical trials have consistently supported the efficacy and tolerability of semaglutide and other GLP‐1 mimetics, making them a preferred treatment option for individuals intolerant to metformin or requiring reinforced management of type 2 diabetes, obesity, and other diseases. Additionally, the flexibility of combining GLP1RAs with other medications allows for personalized treatment approaches to meet individual patient needs.

Furthermore, the drastic increase in the machine learning‐driven algorithms in the scope of computational drug design can also be employed to expedite further the process of target therapeutics development against the GLP‐1 receptor to significantly reduce the side effects of existing drugs on other organs. Yang et al.^97^ employed virtual screening followed by a fluorescent imaging plate reader (FLIPR) based on calcium assay to discover and characterize a novel small molecule GLP1RA called the S6. Similarly, multiple instances of computer‐based molecular docking and dynamic simulations have also been used to identify novel GLP‐1 receptor agonists. Extending this view, it can be anticipated that training ML‐based QSAR models with known GLP1RA data can help predict the biological activity of novel molecules and optimize their chemical structures to enhance potency and selectivity. Similarly, fragment‐based drug design can design novel non‐peptidic therapeutics by integrating the smaller fragments of already available and approved GLP1RAs. By exploiting the insights gained from high‐throughput screening and ongoing trials, non‐peptidic agonists have emerged as a viable avenue for GLP‐1R activation. However, their pharmacodynamics are yet to be fully optimized and can be delved into to design such novel therapeutics.

The increasing number of clinical trials and the subsequent approval of multiple GLP1RAs on the market reflect these agents' growing importance and recognition in diabetes care. Semaglutide, as a frontrunner in this class, has paved the way for improved glycemic control and overall patient outcomes since 2017. In summary, the advent of GLP1RAs has expanded the therapeutic landscape. These medications offer patients various treatment choices, each with its unique benefits and mechanisms of action. As research continues to unravel the potential of GLP1RAs, patients and healthcare providers have access to a range of practical and well‐tolerated options, leading to better management and improved quality of life for individuals with these conditions.

## AUTHOR CONTRIBUTIONS

**Zamara Mariam:** Data curation (equal); resources (supporting); writing – original draft (equal). **Sarfaraz K. Niazi:** Conceptualization (equal); investigation (equal); methodology (equal); validation (equal); writing – original draft (equal); writing – review and editing (equal).

## CONFLICT OF INTEREST STATEMENT

The authors declare no conflict of interest. Pharmaceutical Scientist, Inc. is a professional consulting company in pharmaceutical sciences.

## Data Availability

Not applicable.
